# Current state of smart shunt technology—a future dream or tangible perspective: a systematic review

**DOI:** 10.1007/s00381-026-07509-0

**Published:** 2026-11-04

**Authors:** Ahmed El-Garci, Matthias Schulz, Andreas Schaumann, Gesa Cohrs, Valentina Pennacchietti, Isabel Fernandes Arroteia, Ulrich-Wilhem Thomale

**Affiliations:** Pediatric Neurosurgery, Charité - Universitätsmedizin Berlin, Campus Virchow Klinikum, Augustenburger Platz 1, 13353 Berlin, Germany

**Keywords:** Smart-shunt, Hydrocephalus, Cerebro spinal fluid, Mechatronic shunt valve, Nanogenerator, Sensors

## Abstract

Hydrocephalus, a condition characterized by disrupted cerebrospinal fluid (CSF) dynamics, has historically and mainly relied on ventriculoperitoneal shunt (VPS) systems for treatment. Despite considerable advances in the field, ranging from early valve-less systems to adjustable pressure valves and anti-hydrostatic devices, shunt failure rates remain high, and optimal, individualized CSF drainage remains challenging to achieve. Since hardly any objective feedback measures of shunt function are integrated in the shunt system in order to achieve physiologic like CSF drainage, it somehow remains to be a black box. In response to the aforementioned challenge, the concept of a “smart shunt” has emerged, with the objective of establishing a self-regulating system. The objective of this systematic literature review is twofold: firstly, to analyze existing research on smart shunts; secondly, to synthesize this research in order to highlight any advancements. In order to achieve these objectives, a systematic literature review was conducted in accordance with the PRISMA guidelines, yielding a total of 135 potentially relevant articles. Following the elimination of duplicates and a more thorough evaluation, 22 articles were deemed pertinent for additional scrutiny. Furthermore, a patent search was conducted, which identified ten relevant results. While technological advances render the realization of a smart shunt increasingly feasible, financial challenges remain and further interdisciplinary research and clinical validation are essential to translate this concept into a reliable, personalized treatment option for patients with hydrocephalus.

## Introduction

Hydrocephalus is a clinical and neuroradiographic condition characterized by a complex cerebrospinal fluid (CSF) circulation disturbance [1] and has been recognized and studied for centuries, with historical references dating back to ancient civilizations [2].

The oldest treatment for hydrocephalus consisted of bandaging the head, with written description dating back to the ninth century [3]. Over the course of time, dehydration, strict diets and diuretic medications were also used to treat this condition. However, the benefit of these treatments for patients was only doubtful at best and nonexistent in most cases [4].

With the introduction of CSF-diverting systems into another body cavity, described and realized in 1898 by Ferguson [5] with valve-less shunt systems, the treatment goals started to switch from palliative to curative [6]. The next milestone was reached when valves were integrated into CSF-diverting systems to better regulate the amount of drained CSF. Since then, the therapy of hydrocephalus has been continuously developed with different measures to enhance the quality of shunt implantation surgery (e.g., avoid shunt infections) and a multitude of different valve types to individualize the hydrocephalus treatment modalities [6].

Nowadays, the gold standard for long-term hydrocephalus management is the placement of a ventriculoperitoneal shunt (VPS) and represents one of the most common neurosurgical procedure [7]. Although shunt surgery itself is typically considered a low-risk procedure and has seen advancements in design and management, the rates of malfunction remain to be concerningly high, with approximately 29% of all adults and up to 40% of all pediatric shunt failures within the first year [8] and 60% to 98% of all shunts within 10 years [9–11]. In addition, most shunt patients are likely to undergo at least one surgical revision during their lifetime [12].

Given the fact that an increasing rate of valve systems have included an adjustable functionality and still one of the major factors of long-term shunt failure may relate to uncertainty of how much drainage of CSF the individual patient actually needs, the call for a personalized, self-controlling, and self-regulating shunt system, the “smart shunt,” is recurrently found more often in the literature; however, it has never brought clinical practice. Yet, attention to research and innovation around the topic is still lacking.

The primary aim of this literature review is to systematically analyze and synthesize existing research on smart shunt technology, with a focus on further advancements. This review seeks to identify gaps in the current knowledge and provide recommendations for future research directions. By doing so, the review aims to contribute to a deeper understanding of the current perspective regarding the development of smart shunt systems for the treatment of hydrocephalus.

## Methods

We conducted a systematic literature search according to PRISMA guidelines [13] on PubMed, Embase, Cochrane, and Scopus without time limit using the following combined search terms: “smart shunt,” “shunt” [AND] “smart valve,” “intelligent valve” [AND] “hydrocephalus” [AND/OR] “shunt,” “shunt” [AND/OR] “hydrocephalus” [AND] “wireless,” “artificial intelligence of things” [AND] “human-machine interfaces” [AND] “implantable sensors,” “shunt” [AND/OR] “hydrocephalus” [AND] “neural interfaces,” “hydrocephalus” [AND/OR] “shunt” [AND] “medical technology,” “hydrocephalus” [AND/OR] “shunt” [AND] “closed-loop,” “hydrocephalus” [And/OR] “shunt” [AND] “future direction” [AND] “battery” (Table 1).

**Table 1:** Chronological overview of publications identified through the literature search and their relevance to smart shunt development

Year	First author	Study	Study type	Technology/main focus	Relevance	Functional domain
2007	Sikorski	*Adjustable shunt valve reprogramming at home: safety and feasibility*	Clinical feasibility study	Home-based adjustment of programmable shunt valves	**Direct**	valve adjustment by patient
2008	Momani	*Design of an intelligent and personalised shunting system for hydrocephalus*	Technical/experimental	Intelligent shunt designed for individualized regulation of CSF drainage	**Direct**	Intelligent/adaptive control
2011	Momani	*A mechatronic valve in the management of hydrocephalus: methods and performance*	Experimental	Electronically controlled adaptable hydrocephalus valve	**Direct**	Active valve control
2013	Lutz	*New and improved ways to treat hydrocephalus: Pursuit of a smart shunt*	Review	Development and requirements of smart shunt systems	**Direct**	Smart-shunt concept
2015	Raj	*Demonstration that a new flow sensor can operate in the clinical range for cerebrospinal fluid flow*	Experimental/technical	Sensor for measurement of clinically relevant CSF flow	**Direct**	Flow sensing
2015	Narayanaswamy	*A wireless monitoring system for Hydrocephalus shunts*	Experimental/technical	Wireless monitoring of CSF flow and shunt function	**Direct**	Wireless shunt monitoring
2016	Kim	*Parylene MEMS patency sensor for assessment of hydrocephalus shunt obstruction*	Experimental/preclinical	Implantable MEMS sensor for assessment of shunt patency	**Direct**	Obstruction/patency sensing
2017	Baldwin	*Passive, wireless transduction of electrochemical impedance across thin-film microfabricated coils using reflected impedance*	Experimental/technical	Passive wireless implantable impedance sensing	**Enabling**	Passive wireless sensing
2018	Krishnan	*Epidermal electronics for noninvasive, wireless, quantitative assessment of ventricular shunt function in patients with hydrocephalus*	Translational/clinical	Skin-mounted wireless system for quantitative assessment of CSF flow	**Direct**	Noninvasive flow monitoring
2020	Zhou	*Stretchable piezoelectric energy harvesters and self-powered sensors for wearable and implantable devices*	Review	Piezoelectric energy harvesting and self-powered sensing	**Enabling**	Energy harvesting/self-powered sensing
2020	Krishnan	*Continuous, noninvasive wireless monitoring of flow of cerebrospinal fluid through shunts in patients with hydrocephalus*	Translational/clinical	Continuous noninvasive wireless monitoring of CSF flow	**Direct**	Continuous flow monitoring
2020	D’Antona	*Paradoxical response of intracranial pressure to shunt valve setting adjustments*	Clinical study	Relationship between valve settings and ICP response	**Direct**	ICP-guided shunt management
2021	Srinivas	*Shunt Implants – Past, Present and Future*	Review	Evolution and future development of hydrocephalus shunts	**Direct**	Shunt technology/future concepts
2021	Gamero	*Multimodal Sensing Capabilities for the Detection of Shunt Failure*	Experimental/technical	Multimodal sensing for identification of shunt malfunction	**Direct**	Multimodal failure detection
2022	Lee	*Hydrogel Check-Valves for the Treatment of Hydrocephalic Fluid Retention with Wireless Fully-Passive Sensor for the Intracranial Pressure Measurement*	Experimental/technical	Hydrogel check-valve combined with passive wireless ICP sensing	**Direct**	Valve + wireless ICP sensing
2022	Wang	*Advanced Implantable Biomedical Devices Enabled by Triboelectric Nanogenerators*	Review	Triboelectric energy generation for implantable biomedical devices	**Enabling**	Energy harvesting/power supply
2023	Salih	*Design of a piezoelectrically actuated hydrocephalus shunt valve*	Technical/experimental	Piezoelectrically actuated hydrocephalus shunt valve	**Direct**	Active valve control
2023	Wang	*Artificial intelligence enhanced sensors – enabling technologies to next-generation healthcare and biomedical platform*	Review	Integration of AI and advanced biomedical sensors	**Enabling**	AI-enhanced sensing/processing
2023	Yogev	*Current state of the art and future directions for implantable sensors in medical technology*	Review	Implantable sensors and associated clinical and engineering challenges	**Enabling**	Implantable sensing
2024	Harhangi	*Smartphone-based thermography to determine shunt patency in patients with hydrocephalus*	Clinical/diagnostic study	Smartphone-based thermographic assessment of CSF shunt flow	**Enabling**	Noninvasive shunt patency assessment
2025	Sun	*Moving Toward Human-Like Perception and Sensation Systems—From Integrated Intelligent Systems to Decentralized Smart Devices*	Review/perspective	Distributed intelligent sensing, in-sensor processing, and smart devices	**Enabling**	Intelligent sensing/in-sensor computing
2025	Ohnemus	*CSFsim: A Simulation Framework for Cerebrospinal Fluid Dynamics and Hydrocephalus Shunt Systems*	Computational/modeling study	Open-source simulation of CSF dynamics and hydrocephalus shunt systems	**Direct**	Patient-specific modeling/simulation

*AI* artificial intelligence, *CSF* cerebrospinal fluid, *ICP* intracranial pressure, *MEMS* microelectromechanical systems

*Direct publications* specifically address hydrocephalus shunts, CSF diversion, shunt monitoring or control, or technologies developed for hydrocephalus management. *Enabling publications* describe technologies with potential applicability to future smart shunt systems, including implantable sensing, wireless communication, energy harvesting, artificial intelligence, and in-sensor computing

**Fig. 1:**
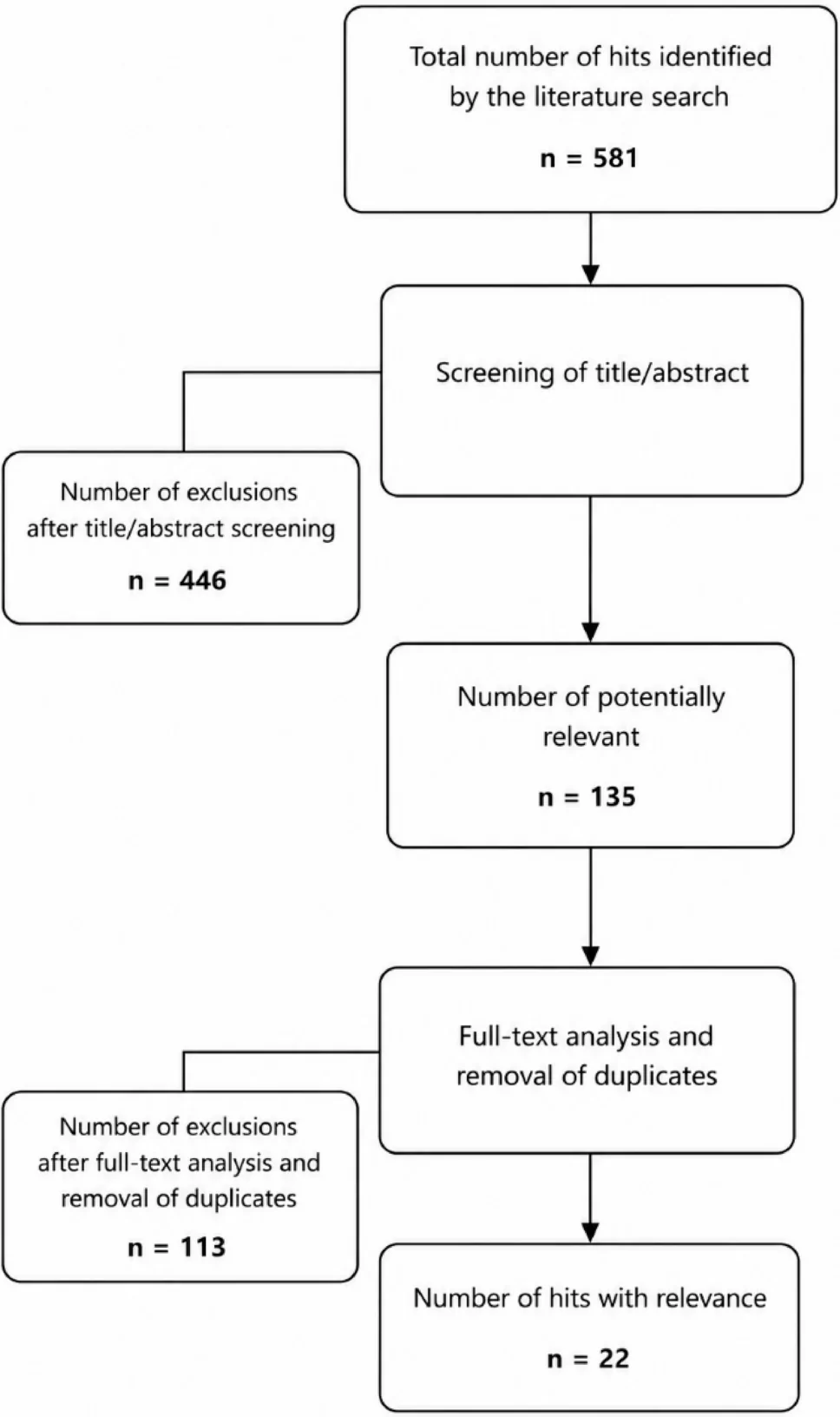
Graphical overview of the literature review

We included all articles relating to shunt systems for the treatment of hydrocephalus, the concept of the smart shunt and its components. Exclusion criteria were as follows: not related to hydrocephalus or its components, cardiovascular shunt, hemodynamic shunt. The search yielded a total of 581 hits, of which 135 remained after first screening for title and abstract and 22 papers remained after removal of non-relevant articles or dublicates. Of the 22 papers examined, four addressed the concept of the smart shunt [14–17], three focused on improving valve technology [18–20], thirteen concentrated on sensors [21–33], one discussed cerebrospinal fluid dynamics [34], and one addressed shunt valve adjustment [35] (Fig. 1).

In addition, a systematic patent search was conducted using the European Patent Office (EPO) database. The following search terms were applied: “intelligent valve” AND “hydrocephalus,” “smart shunt” AND “hydrocephalus,” “smart shunt” AND “valve,” “electromechanical valve” and “hydrocephalus,” “smart” AND “shunt” AND “hydrocephalus.” The preliminary search yielded 282 records. Subsequent to the initial screening and exclusion of patents unrelated to hydrocephalus, 52 records remained. Following a thorough abstract review and the removal of duplicates, a total of 10 patents were selected for inclusion in the final analysis (Table 2).

**Table 2:** Chronological overview and functional classification of patents related to smart cerebrospinal fluid shunt technology

Priority date	Inventor(s)	Patent title	Publication No.	Principal technological concept	Functional classification
07 Apr 1999	Jonas J, Steeger GH	*Device for Normalizing Cerebrospinal Fluid Pressure*	DE19915558A1	Implantable pressure sensor coupled to an electronically controlled flow-regulating valve; programmable target ICP and optional active backflushing	Closed-loop pressure control
20 Jul 2004	Bertrand WJ, Harper DJ, et al.	*Implantable Cerebral Spinal Fluid Drainage Device and Method of Draining Cerebral Spinal Fluid*	US20060052737A1	Implantable CSF drainage system incorporating active pumping and monitoring functions	Active CSF drainage
29 Jun 2007	Ludin L, Mauge C	*Programmable Shunt with Electromechanical Valve Actuator*	US20090005720A1	Adjustable valve, physiological sensor, system controller, and electromechanical valve actuator with external programming capability	Sensor-informed active valve control
19 Nov 2010	Miethke C	*Programmable Hydrocephalus Valve*	DE102010051743B4	Pressure- and optionally posture-responsive electronically actuated valve with processor, data storage, and telemetry	Closed-loop pressure/posture control
13 May 2016	Shachar Y, Chen T, Lobl TJ, et al.	*Skull-Mounted Drug and Pressure Sensor*	US11890111B2	Implantable skull-mounted intracranial pressure sensing and drug-delivery platform	Implantable monitoring / adjunct technology
03 May 2018	Badbada W, Wong NT, Bowman H, Biggers M, Rye J, Shimizu J	*Treatment for Hydrocephalus*	US11464952B2	Shunt technologies including catheter-cleaning mechanisms, anti-obstruction concepts, flow regulation, and electrical monitoring	Shunt maintenance / anti-obstruction technology
22 Dec 2021	Chen TC, Shachar J, Lobl T, Wu W	*Inline Pressure and Temperature Sensor for Cerebral Shunts*	US20230191095A1	Integrated CSF pressure and temperature sensing with wireless transmission and integration with a shunt valve	Sensor-based monitoring and telemetry
05 May 2022	Browd SR, Douglas RJ, Philippsen AOL, Drlik M	*Dynamically Controlled Cerebrospinal Fluid Shunt*	US20230355937A1	Sensor-driven dynamic valve control based on physiological parameters, including pressure-dependent flow profiles and backflushing	Closed-loop dynamic control
06 Jun 2022	Medow J, Iskandar B, Nagy M	*Smartshunt*	US20250352776A1	ICP and posture sensing, microcontroller-based CSF regulation, wireless communication, data logging, rechargeable power supply, and diagnostic functions	Integrated smart-shunt platform
09 Jan 2023	Yang S, Wen P	*Cerebrospinal Fluid Shunting System, Shunting Control Method, and Shunting Valve*	WO2024149147A1	Electronically controlled CSF shunting system combining shunt control algorithms with an actively controlled valve	Automated shunt control

Thereafter, a targeted web search was conducted using Google, guided by the research groups and authors identified through the literature review, to identify ongoing research projects and scientific groups working on smart shunt technologies for hydrocephalus that may not yet have resulted in peer-reviewed publications (Table 3). This yielded four active research groups from the following institutions: the University of Liverpool, the University of Wisconsin, the University of Southern Carolina, and Eidgenössische Technische Hochschule Zürich.

**Table 3:** Research groups involved in ongoing smart shunt research identified through the targeted web search

Institution	Country	Research activity identified
University of Liverpool	UK	Ongoing research on smart shunt technologies, including the conceptual development of a theoretically mechatronic shunt valve
University of Wisconsin	USA	Investigation of switching and control algorithms aimed at improving the regulation and performance of closed-loop shunt systems
University of South Carolina	USA	Research on smart shunt technologies, including the development and evaluation of individual components that may contribute to future integrated smart shunt systems
ETH Zurich (Swiss Federal Institute of Technology Zurich)	Switzerland	Development of a smart shunt valve prototype intended for further evaluation in an animal model study

## The past and presence of shunt valves

**Fig. 2:**
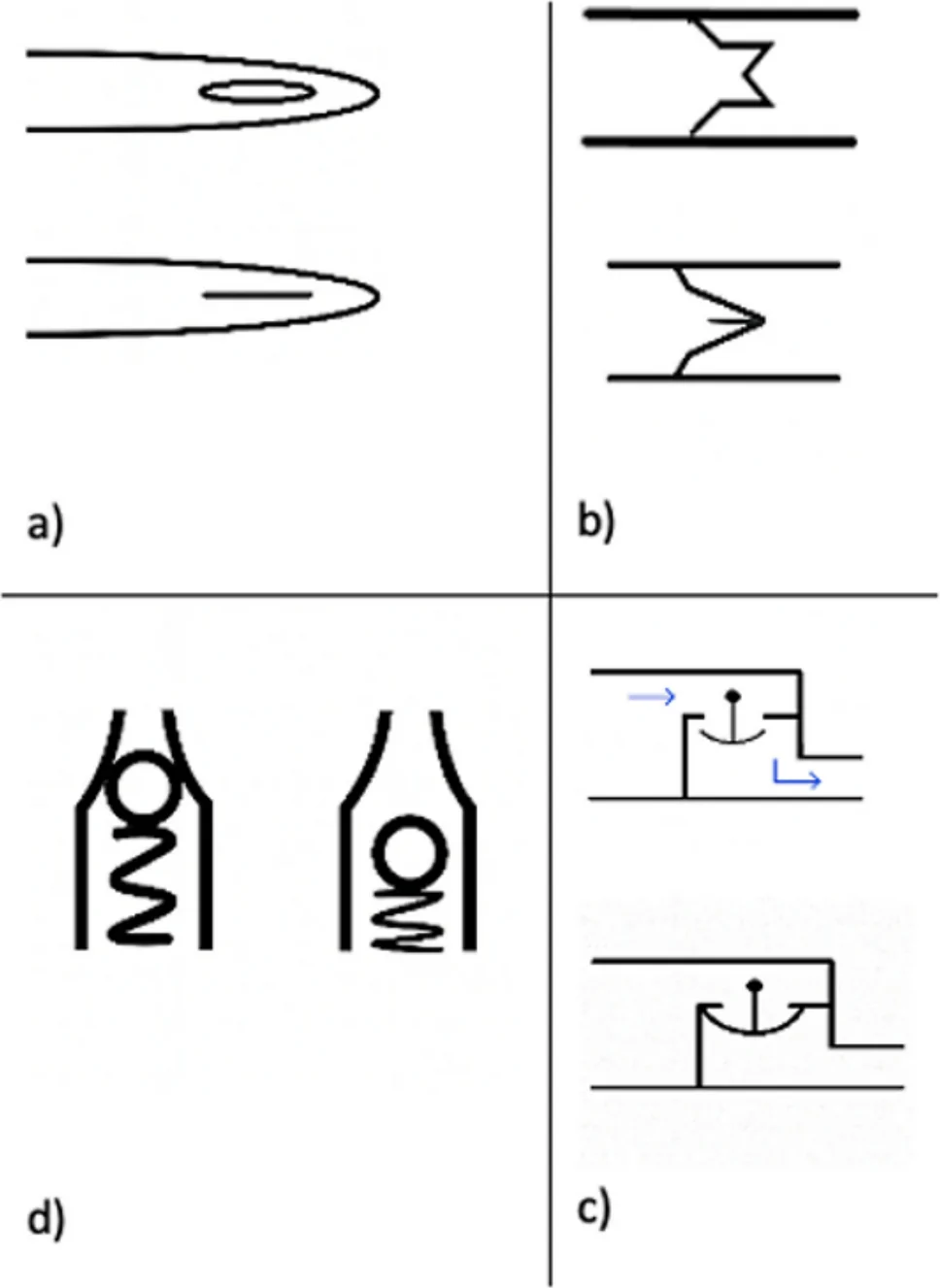
**a** Slit valve: Unidirectional CSF flow is regulated through a pressure-dependent slit, with the opening pressure determined by the geometry and mechanical properties of the valve material. **b** Mitral (duckbill) valve: Increasing CSF pressure separates two opposing leaflets to permit drainage, while decreasing pressure allows the leaflets to return to their resting position and close the valve. **c** Ball-in-cone valve: CSF pressure exceeding the predefined spring tension displaces the ball from the cone, permitting CSF drainage until the pressure gradient decreases and the valve closes. **d** Diaphragm valve: Increasing CSF pressure deforms or displaces the diaphragm to open the valve, which closes again when the pressure falls below a predefined threshold. Adopted from Drake JM, Sainte-Rose C. The shunt Book. Cambridge, Mass: Blackwell science; 1995

One of the most frequent complications associated with the implantation of shunts lacking any valve system is CSF over-drainage and the possible formation of blood clots or tissue segments sucked secondarily into the shunt, which can be attributed to an absence of cerebrospinal fluid flow control [36]. That could be ameliorated after introducing shunt valves into CSF-diverting systems. In the mid-1950 s, the first mechanical valves were developed, and four distinct construction designs (ball in cone, slit, diaphragm, and mitral) (Fig. 2) stood out and became the cornerstones of later developments [37].

### Technical evolution of shunt-valves

The first valves acted with a defined differential pressure between the proximal shunt part towards the ventricular system and the distal shunt part towards the CSF draining cavity. Often, three different pressure settings (low, medium, high) were manufactured to be chosen for the individual patient. A pressure change, if needed, would be associated with a new surgery in order to exchange of the valve itself.

Despite this significant advancement, over-drainage persisted as a prevalent complication, occurring in approximately 55% of cases [38, 39] with still a high rate remaining unidentified due to a lack of awareness towards this problem. The development of adjustable differential pressure valves and, secondly, the integration of over-drainage protection units was the consequent step of advancement.

### Over-drainage protecting units

In his 1973 scientific paper, John L. Fox investigated the influence of CSF shunts on CSF dynamics and intracranial pressure and was able to prove that in the upright position, negative hydrostatic pressure has a relevant influence on the differential pressure valve and can therefore directly influence the CSF flow rate [40]. In the same year, Harold Portnoy and Rudolf Schulte presented the first viable concept of an anti-hydrostatic valve, which was able to counteract the enhanced flow of CSF in upright position by means of a diaphragm. As soon as the pressure distal to the valve became negative, the membrane was supposed to occlude the outflow [41]. The technology is available as Delta chamber (Medtronic). In addition to valves with a membrane function, a ball-in-cone variant was also developed simultaneously, acted by means of gravity with a ball falling into the cone outlet of the unit, blocking CSF flow, and increasing the valve’s resistance in vertical position. The combination of fixed DP valves and fixed gravitational units was reported to reduce the annual risk of proximal catheter obstruction, as a sign of over-drainage, by up to 92% by Gruber et al. [42]. Furthermore, the risk of over drainage–related complications such as low-pressure headaches, subdural hygromas/hematomas, slit ventricles, and acquired Chiari I Malformation was reduced by up to 84% as reported by Gruber and Roehrig [43]. Gravitational units are available from many different companies such as the Shunt Assistant (Aesculap-Miethke, Germany), the Z Flow valve (Chhabra, India), the anti-siphon device (Integra, USA), and the siphon X (Sophysa, France) [44]. Another overdrainage protecting unit is the Siphon Guard (Codman-Integra, USA), which would close its regular sized middle CSF tube in high-flow conditions diverting it into a spiral with lower diameter and increased length and thereby temporarily increasing the units resistance according to the Hagen-Poiseuille’s law [45].

### Adjustable differential pressure valves

Despite the substantial reduction in shunt-related complications that was achieved through the introduction of over-drainage protection devices, the ongoing necessity to enhance clinical outcomes led to the development of adjustable differential-pressure valves. The first implanted adjustable differential pressure valve was developed by Kuffer and Strub in 1969 and consisted of a piston that compressed a spring. By adjusting the piston with a screwdriver, the tension of the spring and thus ultimately the pressure resistance could be adapted [46]. The fact that the pressure could only be changed intraoperatively may have contributed to its failure for wider recognition. However, 4 years later, Salomon Hakim presented a percutaneously adjustable shunt valve that stood out due to its precision and evolved as a huge success and finally achieved worldwide application [47]. Modern adjustable differential pressure valves usually feature a ball-in-cone mechanism with spring-variable tension that can be adapted non-invasively using a magnetic field transmitting device through the skin to adjust the position of the spring [6]. The range and number of steps of adjustments do vary among the different manufacturers. The integration of MRI imaging into everyday clinical practice led to the development of MR-compatible valves, which did not change their setting due to arbitrary external magnetic force alone (e.g., proGAV (Miethle-Aesculap), Polaris (Sophysa), StrataMR II (Medtronic) and CertasPlus (Integra)).

Consequently, shunt valves combine the different regulating products with both an adjustable differential pressure unit and a fixed over-drainage protection unit (e.g., Codman Hakim valve with a Siphon Guard (Integra), proGAV including a Shunt Assistant (Miethke-Aesculap), Strata valve with a Delta chamber (Medtronic)).

Quite recently, an adjustable gravitational valve was added as additional valve type in which the resistance only in vertical position can be adjusted accordingly (e.g., proSA, M.blue (Miethke-Aesculap)). The PROSAIKA study, published in 2015, demonstrated that the implantation of an adjustable gravitational valve resulted in an 86% alleviation of hydrocephalus symptoms as reported by Kehler et al. [48]. This valve may also be combined with an adjustable differential pressure unit giving the possibility to adapt the opening pressure in all body positions individually (e.g., M. blue plus (Miethke-Aesculap)) [8].

## The smart-shunt concept

The CSF-diverting shunt system however remains to be a “black box” giving no reliable information about the amount of CSF drainage as well as the actual intracranial pressure resulting from its treatment. Indirect measures such as clinical condition and ventricular size evaluation from different imaging modalities are used in daily clinical practice but remain limited in application to optimally guide the patient towards their individual needs. In this context, a “smart shunt,” defined as an implantable CSF diversion system that integrates sensing, processing, and actuation to dynamically adapt CSF drainage to the individual patient’s physiological requirements, by establishing a feedback-controlled, ideally closed-loop system [14, 49], appears to be a reasonable further step of development. Possible expectation for such a valve system would be to detect changes in cerebrospinal fluid dynamics in the system and to automatically adapt those to the patient needs. To enable this, integrated sensors are required which might monitor the ICP and/or CSF flow rate. Controlling the patency of the shunt catheters would be a simplified feedback method to control the shunt function in general. Being able to measure the CSF flow would allow a more detailed analysis of individual patient’s needs, while identifying the ICP in a shunted patient is giving the target parameter for treatment since the basic purpose of the CSF-diverting shunt system is to normalize the intracranial pressure. The theoretical capacity to acquire both measures from externally, as well as the ability to make changes to the valve setting in order to titrate the measurements towards most physiological parameters, seems to lead towards the smart shunt concept. An automated feedback-controlled, closed-loop system would enable internally measured parameters to be regulated by changing the CSF flow aiming for the optimal ICP at any given moment in the individual patient. The realization of such a shunt necessitates not only the technology for sensor measurements and automated adjustment options but also the utilization of a sufficiently powerful battery. Concurrently, the battery must be sufficiently miniaturized as well as be capable to last an adequate period of implantation time.

### Sensors

Contemporary sensors implemented in health care have become more and more sophisticated over the past decades and are generally capable of detecting changes in pressure, voltage, temperature, light, and other parameters [27]. The integration of these sensors in health care application can be used as external “add-ons” used for diagnostic purposes or to modulate implanted systems, for example in the form of a wearable device. In addition, sensors integrated in implanted systems enable mainly modulating purposes over long periods of time.

#### Wearable sensors

The benefit of wearable sensors lies in the simpler and more cost-efficient production, which is due to the elimination of the biocompatibility, miniaturization, and surgery required for implantation [50]. Non-invasive sensors may be, however, inferior to implantable sensors with regard to measurement accuracy and may be limited in measuring bio-signals that can only be extrapolated through the skin [27]. Nevertheless, also due to their flexible applicability, a growing trend in investment is evident in the development of non-invasive sensors and wearables, with forecasts indicating that their economic growth may exceed that of implantable sensors by 2030 [51, 52].

With regard to the present topic of hydrocephalus treatment, an external sensor that can possibly provide information about shunt function was introduced in 2018 by the Department of Electrical Engineering at Stanford University. Its purpose was to identify CSF-flow through the shunt tubing by applying heat to the skin above the shunt catheter. It consists of a heatable diode and a temperature meter, which was placed along the shunt [26]. The temperature changes distal to the diode along the subcutaneous tubing would indicate positive flow or no flow. Quantitative measures, however, are not applicable. The same principal was presented by Harhangi et al. who used a cool-pack and a thermal imaging camera to determine CSF flow through a shunt catheter with a specificity of 88%. [32]

As early as 2016, the company Brain4Care introduced a wired headband sensor device. A micro-expanding sensor can detect skull oscillations that correlate with the heartbeat frequency. These oscillations can be detected as a waveform that is basically similar to an ICP curve, but without direct measurement. The curve morphology changes, however, give indirect information about the intracranial compliance, which may possibly be translated into certain ICP segments. Subsequent developments led to a fully wireless version of the device, which was approved by the FDA in 2021 [53]. The device is recommended to be used for diagnostic purposes, e.g., for screening purposes and for modulating hydrocephalus treatment or to monitor the shunt function indirectly, in uncertain conditions [53].

#### Implantable sensors

A multitude of sensors have become pivotal implantable component in medicine within contemporary clinical practice in evolution towards progressively precise physiological recordings. A discernible economic trend is also emerging, with a compound annual growth rate (CAGR) of 11.1% for the implantable sensor market from 2020 to 2027 [51]. The ongoing miniaturization of implantable sensors, driven by continuous technological advancement, has enabled their integration into compact systems, facilitating their use in a wider range of applications (e.g., self-regulating diabetes pumps, ECG measuring heart pacer, EEG detecting vagus nerve stimulator) [27]. Additionally, there is an emerging trend towards intelligent systems incorporating machine-learning capabilities, with in-sensor computing offering the potential to reduce response latency while improving computational and energy efficiency [33].

In the context of hydrocephalus management, distal flow rate measurements have been demonstrated to be feasible by means of implanting and connecting sensors into the distal catheter. The data obtained can be used to exclude occlusion and to measure the amount of CSF drained over time [23, 24]. Another approach to occlusion detection was published in 2016 and presents a sensor that has electrodes both on the inner and outer wall of the ventricular catheter. The sensor can detect occlusion of the ventricular catheter by measuring impedance changes. Yet, its sensitivity highly depends on the location of the occlusion with respect to the sensor’s position and might not be relevant in case of distal shunt failure [21].

Thus, the presented sensors are still ineffective and will be unable to determine the location of dysfunction (proximal vs. distal) and will be not relevant to possibly regulate the shunt function itself. A new approach was introduced by Arul Narayanaswamy who placed multiple pressure sensors along the shunt catheter, which may detect pressure increase proximal to an occlusion [25].

The development and miniaturization of microtransducers in the early 2000 s resulted in the creation of sensors for measuring intracranial pressure (ICP), which superseded the previous analogue measurement of the water column. The production of these sensors was primarily undertaken by three major companies (Camino, Codman, Raumedic), and their utilization was restricted to patients necessitating in-hospital mostly neurointensive monitoring [54]. However, the advent of implantable pressure sensors with telemetric data transmission capability marked a significant milestone, enabling outpatient ICP monitoring as well. In the last millennium, seminal contributions were made to this field by Zervas [55] who implanted the first telemetric ICP sensor, by Chapman [56] who used telemetric ICP sensors for the first time in a postoperative setting, by Inagaki [57] who helped develop microtransducers for these telemetric sensors and by Richard [58] who evaluated first the clinical outcome of hydrocephalus treatment with an integrated telemetric ICP sensor. In the subsequent two decades, Raumedic and Miethke have advanced the state of the art in telemetric ICP measurements with their sensors, Raumedic Neurovent P-tel (2009) [59] and Miethke M.scio (2015), respectively [60, 61]. The two sensors (Raumedic vs. Miethke) differ in several key specifications. Firstly, the location of the measuring sensor differs, with the sensors positioned parenchymal (Neurovent P-tel) or within the CSF filled implant with a connection to the ventricular system (M.scio), which results in the fact that the Neurovent P-tel contrary to the M.scio device would need to be placed with an additional surgical incision, while the M.scio can be integrated in a shunt system or reservoir as stand-alone implant [60, 61]. Secondly, the durability of the sensors differs, with the Raumedic sensors reported to last 3 months while the Miethke sensors are licensed to endure for longer years. Both sensors are activated and read out using a radio-frequency identification (RFID) reader.

A US patent filed in 2016 for a smart intrathecal drug pump details the application of a spectrographic sensor in conjunction with a pressure gauge for the targeted determination of drug concentration and ICP monitoring and automatic dose adjustment [62]. This system holds particular significance for oncological patients, but a similar solution might also be employed as an active valve in a VP shunt in hydrocephalus patients by connecting it to a draining catheter [62]. In the ensuing year, another US patent was filed for a combination of sensors suitable for integration into a shunt system, thereby converting it possibly into a smart shunt [63]. Utilizing induction energy, the sensors would be activated and data on the pressure, flow rate, and composition of the cerebrospinal fluid read out [63]. However, no reports of commercialization of this presented system are published so far.

#### Power supply

Power supply to smart implants constitutes a pivotal consideration in the development for long-term implantation. Three distinct approaches are defined in this respect. These include power storage (e.g., batteries, supercapacitors), power generation (e.g., piezoelectric, friction electric energy harvesters) and wireless charging.

Since the first implantation of a cardiac pacemaker in 1958, battery technology has undergone rapid development. Following the inception of nickel-cadmium accumulators, which exhibited a lifespan of merely a few hours, a substantial transition has ensued towards mercury-zinc batteries [64, 65]. Despite their capacity for higher energy output and extended service life, these batteries resulted in the production of hydrogen during the discharge process. This phenomenon compromised their usage for implants, leading to possible leakage of toxic substances [66]. Following the introduction of radionuclide batteries in the 1960s, which provided electricity through thermoelectric conversion, the issue of hydrogen production was superseded by that of radiation exposure. These batteries were typically fueled with plutonium-238 and were capable of supplying the system with electricity for several decades without requiring replacement [67]. Despite their proven reliability, these batteries were gradually withdrawn from the market around a decade later due to significant radiation-related safety concerns [68]. Concurrently, lithium iodide batteries were patented in 1972, delivering a constant voltage, with no radiation or toxic substance emissions, and a life expectancy of about 10 years [64]. These attributes have ensured their continued utilization also in health care sciences to the present day. These technological advancements have established lithium batteries as the predominant power source for implantable devices on a global scale [69].

The utilization of nanogenerators as an interesting alternative to primary batteries is a subject that is currently being increasingly investigated [27–29, 70]. The function of these nanogenerators is to cover the power consumption of implanted systems by means of mechanical-electromagnetic coupling. Generators that utilize pressure to generate electricity are designated as piezoelectric nanogenerators (PENGs) and have already demonstrated the capability to generate voltages of up to 5 V and currents of 700nA [70]. In a particular instance, the PENG was implanted in a pig’s heart, where it successfully converted every heartbeat into electrical voltage [70]. In addition to pressure, friction can generate electricity under the principle of the triboelectric effect. Those triboelectric generators (TENGs) have been shown to achieve a maximum voltage of up to 55 V and a current of 0.6 µA following implantation in the dorsal subcutaneous tissue of a rat [71]. However, biocompatibility plays an essential role in the development of both PENGs and TENGs, with one of the main focuses currently on bioabsorbable materials [72], and it remains to be proven if generated energy will be sufficient to cover the need of a smart valve system.

As for the wireless power supply, it can be used either intermittently to charge a battery-equipped sensor or directly apply energy to actuate a sensor, with inductive near-field coupling being the primary method employed [64]. In cases involving deeper tissue implantation, ultrasound, electromagnetic waves or magnetic fields are typically utilized [27].

#### Mechatronic valve scheme

In addition to component miniaturization, biocompatibility, and power supply, the software that controls a mechatronic shunt valve is essential to creating a feedback-controlled CSF-diverting system. A smart valve may be regulated either via an open-loop protocol (predefined timing CSF flow), a closed-loop (sensor feedback CSF flow), or an “intelligent” closed-loop with patient and healthcare professional intervention options with adaptable sensor feedback thresholds for CSF flow.

**Fig. 3:**
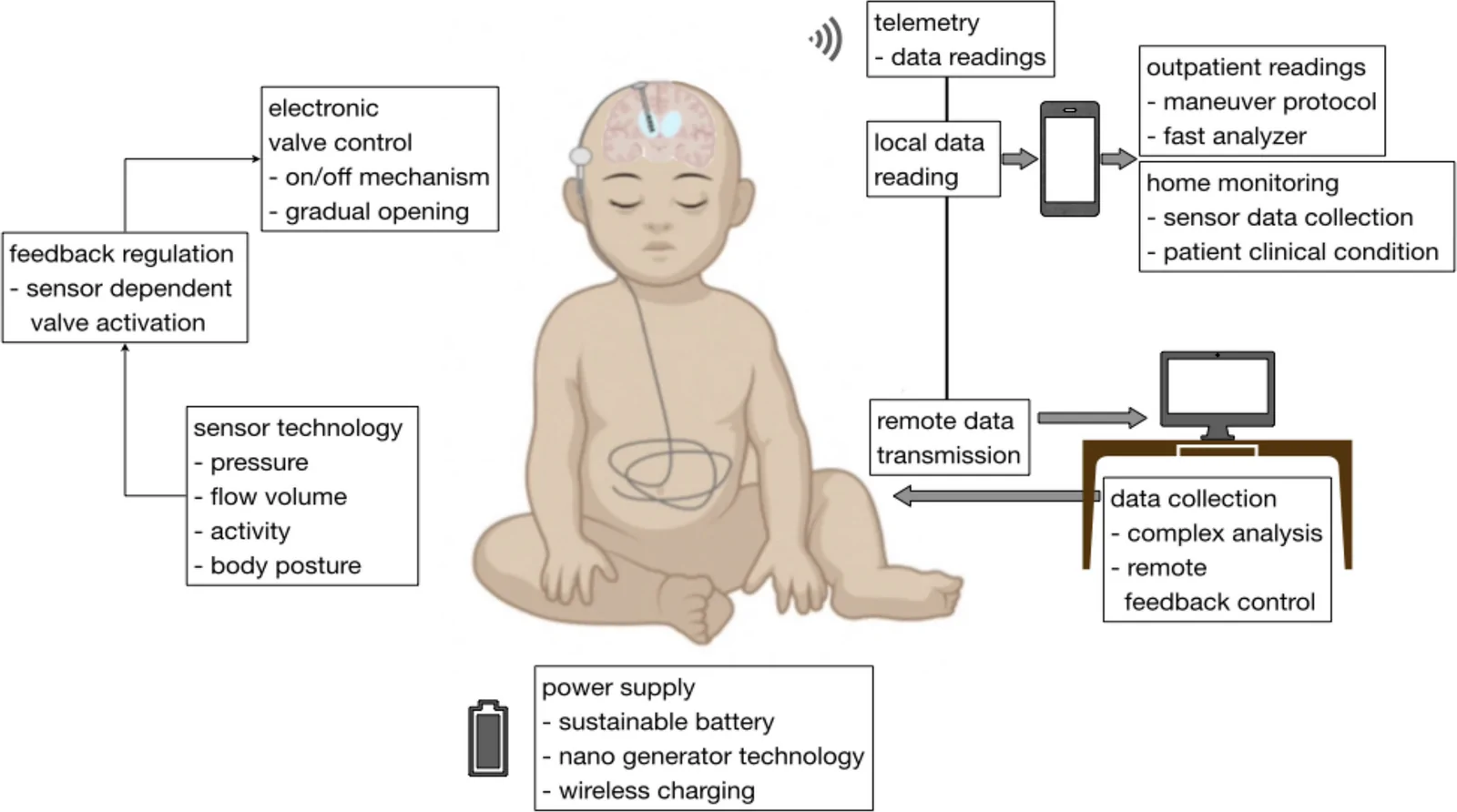
Schematic visualization of a smart shunt concept. Left: Control algorithm for the mechatronic valve with an open (top), closed (middle), and intelligent closed-loop (bottom). Bottom: Power supply possibilities. Right: Telemetric data transfer and evaluation

The open-loop is a timer mechanism that, e.g., opens and closes the valve at pre-determined time interval for pre-determined duration (Fig. 3). An experimental in-vitro study has shown that with an opening frequency of 1/20 per minute with an alternating opening duration of 5 min (during which the valve is opened and closed every minute), the ICP can be kept in a physiological range compared to a conventional mechanical valve if the opening algorithm meets the needs of individual hydrocephalus treatment [73]. If the interval between openings or the opening duration is too long, respectively, underdrainage or overdrainage may occur in a given setting [73]. However, the intracranial pressure dynamic conditions of each patient and interpersonal needs for CSF flow according to the underlying CSF pathophysiology may more likely vary widely; thus, individualized adjustment of the CSF flow may be critical to the success of shunt therapy.

Thus, the utilization of closed-loop feedback facilitates the adjustment and adaptation of the opening frequency and duration in relation to the detected ICP condition, thereby aiming for the maintenance of intracranial pressure within normal range on a more individualized basis and enabling physiological drainage. This process is facilitated by the system’s capacity to analyze ICP data, obtained from ICP pressure sensor integrated within the system [74].

However, the implementation of a closed-loop configuration poses numerous inquiries, including the optimal measurement frequency of ICP changes, the type and variability of valve adjustments defining the CSF flow activation, the associated material needs, and the power consumption. Intelligent control mechanisms are expected to be not only self-regulating but also to provide medical staff and patients with the opportunity to participate in the evaluation and adaptation of the regulatory circuit [15, 17]. The integration of pressure and flow sensors in conjunction with communication modules will monitor the actual profiles of ICP and CSF flow at a given time point as feedback data. That will enable to adapt thresholds of external valve activation and might also provide timely notifications to patients and medical staff in the event of possible shunt failures (e.g., occlusion detection). Furthermore, the implementation of advanced algorithms has the goal to achieve physiological intracranial CSF pressure dynamics on an individualized basis and even to facilitate possible weaning procedures from a shunt system especially in cases if internal reparation processes might lead to normalization of CSF dynamics over time [14].

#### Recent research achievements

As early as 1988, Harold Rekate presented a comprehensive theoretical model of a smart shunt which, in addition to measuring flow rate and regulating the shunt-valve autonomously, would also be capable of communicating with medical staff via a modem technology (e.g., cerebrospinal fluid control). However, the realization of such a system was precluded by the limited technological capabilities of the era. Towards the close of the twentieth century and the advent of the twenty-first, three patents were filed, delineating sensor-controlled mechatronic shunt valves designed to autonomously regulate cerebral CSF drainage [75–77]. Two of these concepts incorporated the option of an integrated pump, thereby enabling active rather than exclusively pressure-driven CSF drainage [75, 76].

In the early 2000s, the University of Liverpool group, led by Prof Waleed Al-Nuaimy, presented theoretical smart shunt ideas in which a system was proposed which connects to the internet to communicate remotely with medical staff [15, 78]. This would have enabled any dysfunction to be recognized at an early stage. It was even part of the vision that the pressure setting of the valve could be changed remotely by the healthcare providers [79]. In addition, proposals for the operation of an open-loop system were presented, which would be controlled as a time switch device [73].

In 2018, Prof. Bermans Iskander’s research group at the University of Wisconsin presented a laboratory prototype of a smart shunt that uses an ICP pressure probe and an intelligent closed-loop algorithm. This prototype is reported to be capable of disregarding short-term ICP peaks, focusing on the mean value and thus preventing unnecessary drainage of CSF if ICP is kept in normal range. Furthermore, the system was supposed to have the capacity to communicate with a handheld device, thereby possibly facilitating the early detection of complications and dysfunctions to enable timely intervention. An experimental animal study is currently planned but has not yet been published [80]. Contemporaneously, Miethke and Shachar independently filed patents that described closed-loop smart shunt concepts integrating a mechatronic valve, pressure and position sensors, and telemetric data transmission within a single housing. However, Shachar’s system was primarily conceived as an intraventricular drug-delivery device, with the option of functioning as a smart CSF shunt when connected to a distal peritoneal catheter [62, 81].

Also in 2018, the University of Southern Carolina, under the direction of Professor Ellis Meng, presented several smart shunt components (e.g., pressure and flow rate sensors). According to the institution’s own information, all components have already been manufactured [82] and have partially been tested in vitro [21]. Despite extensive research, no publication on a completed prototype is available yet. However, a patent by Badbada et al. described a smart shunt system that, in addition to a mechatronic valve and pressure and gyrational sensors, incorporated an active occlusion-clearing mechanism designed to restore shunt patency in the event of obstruction [83].

Subsequent years have seen the filing of further patents describing self-regulating, intelligent closed-loop shunt systems, incorporating multiple sensors and, in some cases, the capability to transmit physiological or device-related data to the treating team and/or the patient with the aim to enable a more individualized and adaptive treatment of hydrocephalus [84–86].

The ETH Zurich (Eidgenössische Technische Hochschule Zürich) unveiled the VIE-shunt (ventricular intelligent and electromechanical shunt system), a smart shunt prototype that incorporates a closed-loop control element with a micropump, an inertial sensor, a flow rate sensor, and a pressure sensor. The compact size of the components is supposed to enable their integration within a single housing. According to the company, the subsequent phase involves the feasibility and efficacy testing in a sheep model [87]. With the exception of the VIE-Shunt, no evidence of prototype development could be identified for the other patented systems.

## Discussion

The commercial production of a smart shunt would represent a paradigm shift in the therapeutic management of hydrocephalus, with autonomous and individualized shunt regulation becoming the new standard. However, before this can be achieved, the development of an intelligent algorithm is necessary. This algorithm would need to analyze the information from the sensors and adjust the valve in order to maintain the ICP and CSF circulation within physiological parameters. The algorithm must be capable of recognizing the patient’s individual adaptation capacity and needs, accordingly. Furthermore, the shunt may be designed to provide early warning in the event of detected malfunction, thereby helping to avert potential emergency or hazardous scenarios. A marked increase in the number of scientific publications on this topic is indicative of its growing interest [14, 88–90].

However, the question of power supply is also an essential part of the topic and, owing to the incredible technological advances and component miniaturization in recent decades, offers unprecedented possibilities, ranging from wearable and implantable self-powered sensors to nanogenerators. Yet, the measurement type and rate of the sensors, in conjunction with the mode of operation (intermittent vs. continuous measurement) and the mode of possible external adaptation, will be essential in determining the capacity of power supply [27, 28, 71].

A further factor that must be considered is the financial aspect of such a system. In addition to the resources required for its development, a significant budget and time are necessary for its development and final approval as a medical product. In Europe, the approval process can take over a year and can require an investment in excess of a relevant amount expenses [91].

Furthermore, the question of reimbursement by the health care system requires further elucidation of possible advantages of such a smart shunt in comparison to existing mechanical shunt systems. The global VP shunt market, with an anticipated compound annual growth rate (CAGR) of 6.5% and an equally anticipated market size of USD 600 million by 2033 [92], signifies an immensely lucrative market that is propelling and driving research and additional development in this domain [14]. However, better reimbursement models are yet to be defined. Close interaction of the stakeholders (patient representatives, neurosurgeons, medical device industry) is necessary to increase the lobby of hydrocephalus as a chronic disease which still needs significant improvement of care.

## Conclusion

In the light of the technological advances that have been made in recent years and ameliorated comprehension of hydrocephalus pathophysiology, a development and subsequent commercialization of a smart shunt seems to be a more viable prospect than ever before. While further experimental and clinical investigations are required to further validate these findings, it is hypothesized that shunts will gradually evolve into smart shunts, with the eventual presentation of more sophisticated shunt systems in the foreseeable future.

## Data Availability

No datasets were generated or analysed during the current study.
